# Modification and coupled use of technologies are an essential envisioned need for bioaerosol study – An emerging public health concern

**DOI:** 10.1016/j.fmre.2021.10.012

**Published:** 2022-01-10

**Authors:** Prakriti Sharma Ghimire, Lekhendra Tripathee, Shichang Kang

**Affiliations:** a State Key Laboratory of Cryospheric Science, Northwest Institute of Eco-environment and Resources, Chinese Academy of Sciences (CAS), Lanzhou 730000, China; b Himalayan Environment Research Institute (HERI), Kathmandu 44602, Nepal; c University of Chinese Academy of Sciences, Beijing 100864, China

**Keywords:** Bioaerosols, Environment, Bioaerosol measurement, Public health concern, Airborne microbiology

## Abstract

The airborne microbiome is one of the relevant topics in ecology, biogeochemistry, environment, and human health. Bioaerosols are ubiquitous air pollutants that play a vital role in the linking of the ecosystem with the biosphere, atmosphere, climate, and public health. However, the sources, abundance, composition, properties, and atmospheric transport mechanisms of bioaerosols are not clearly understood. To screen the effects of climate change on aerosol microbial composition and its consequences for human health, it is first essential to develop standards that recognize the existing microbial components and how they vary naturally. Bioaerosol particles can be considered an information-rich unit comprising diverse cellular and protein materials emitted by humans, animals, and plants. Hence, no single standard technique can satisfactorily extract the required information about bioaerosols. To account for these issues, metagenomics, mass spectrometry, and biological and chemical analyses can be combined with climatic studies to understand the physical and biological relationships among bioaerosols. This can be achieved by strengthening interdisciplinary teamwork in biology, chemistry, earth science, and life sciences and by sharing knowledge and expertise globally. Thus, the coupled use of various advanced analytical approaches is the ultimate key to opening up the biological treasure that lies in the environment.

## 1 Introduction

The global interest in bioaerosols has rapidly increased to widen the awareness of their distribution, characterization, quantification, and health impacts (e.g., respiratory diseases, allergies, infectious diseases, and cancer). In recent years, studies on exposure to bioaerosols in both occupational and residential environments have provided data about the probable impacts on human health, showing the benefits and harmful effects of bioaerosols. However, correctly describing their role in the origination or deterioration of diverse symptoms and diseases remains problematic. The widespread distribution and nature of bioaerosols followed by their survival in the atmosphere are the major issues of concern that can shape the understanding of their risk to human health. In 2015, International Astronautical Federation (IAF) publishers printed a conference paper presented at 66th International Astronautical Congress 2015 [1]. The article mentioned standard small satellite architecture for space microbiology. The description provides a vision toward developing cost-effective, space-based microbiology research, especially for university students and professors. Although the research obstacles have not been minimized yet, the concept has opened an alternate method for bioaerosol studies, especially in the real-time aspect of attaining an accurate measure of microbial particles present in the air at a specific time and area.

**Fig. 1:**
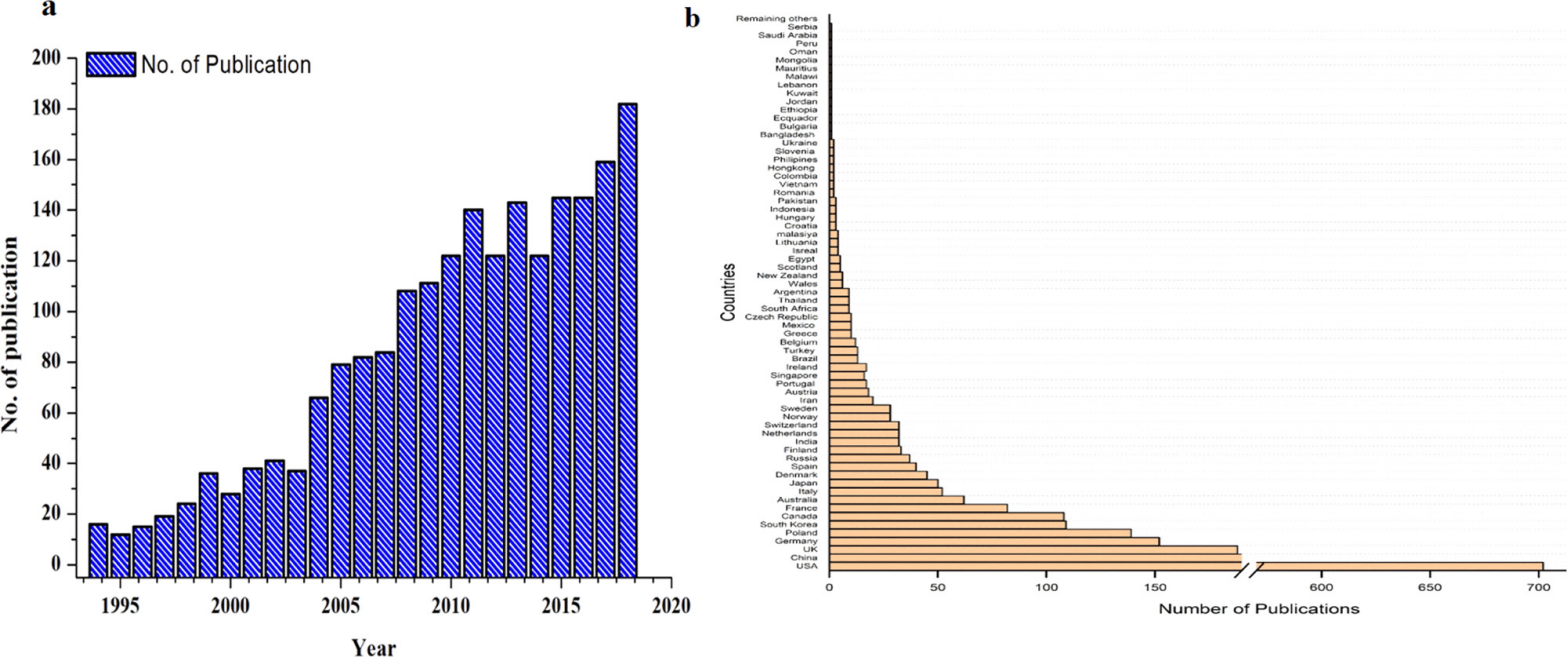
**Global representation of statistics of bioaerosol study performed all over the world.** (a) The number of publications counts on bioaerosol study (1997-2018). (b) Global statistics of bioaerosol publication from different countries all over the world. The color legend shows the number of publications. The data were obtained from the web of science database search by using bioaerosols as the keynote.

As illustrated in Fig. 1, interest and research in bioaerosol studies have increased significantly over the last two decades. Indeed, in comparison to other studies on the environment and pollution, bioaerosol analysis is unlikely to have garnered much attention today. However, some significant steps taken for the analysis of microorganisms present in the air have raised interest in the study of bioaerosols in recent decades [2]. Identification of specific microorganisms or microbial communities in the atmosphere, particularly in the outdoor environment, is a tough but necessary undertaking. It is believed that airborne particles (both physical and biological particles) contain large amounts of information about the environment and microorganisms and their impact on humans and climate change [3]. Several factors impede the risk assessment of bioaerosols, such as the complexity of microorganisms, the techniques of sampling, and the lack of valid quantitative criteria (*e.g*., exposure standards and dose/effect relationships) [4]. Exposure to some microbes is thought to be beneficial for health, but additional research is necessary to appropriately assess their potential health hazards, such as infectious capabilities, dormant nature, interindividual vulnerability, interfaces with nonbiological agents, and some other proven/unproven health effects (*e.g*., atopy and atopic diseases). Thus, the primary objective of this article is to provide an overview of the global state of bioaerosol research in terms of pre-existing knowledge, different methodologies adapted for bioaerosol measurement, the current state of technology, and significant advances in bioaerosol quantification, detection, and characterization research. Furthermore, this short review provides perspectives on bioaerosol research progress and limitations and scrutinizes vitally necessary research techniques that include multidisciplinary collaboration. This article provides brief information on some of the sensitive and effective methods for bioaerosol analysis and the necessity of the use of some cross-disciplinary techniques to better understand the biology of air.

## 2 Some successful cutting-edge descriptions of aerosol microbiology

Although Charles Darwin was the first scientist to discover the transport of dust particles in the air, Louis Pasteur initiated methods of bioaerosol sampling and microbial research in the air [4]. However, because all microbes could not be cultivated on a culture plate, several microbes were not observed until DNA-based experiments were established. Following this discovery, many researchers have identified copious microorganisms in the air using modern and advanced tools.

The use of different samplers has provided considerable variation in microbial detection. Bowers et al. (2013) identified the composition of airborne communities with variabilities based on size, season, and duration using coarse (PM_10 −2.5_) and fine (PM_2.5_) quartz filters [5]. A comparative study of different samplers and analysis methods was performed by Xu et al. (2011). They demonstrated that the BioStage impactor, BioSampler, and MCE filter are power pact samplers for culturable biological particles, such as *Alternaria, Cladosporium*, and *Aspergillus* [6].

Miaskiewicz-Peska and Lebkowska (2011) performed a study on filter efficiency by using two woven air filters: P2 and P3 (Secura B.C., Warsaw, Poland). The experimental setup used the PALAS set (PALAS GmbH, Karlsruhe, Germany) conducted with mineral aerosol ISO Fine Test Dust, Model 12103-1-A2 (Powder Technology Incorporated, USA). The results showed that 100% filter membrane efficiency could not be achieved when nonbiological aerosol filters are used to collect biological particles present in the air. However, some potent aerosol microorganisms, such as *Micrococcus luteus, Micrococcus variants, Pseudomonas putida,* and *Bacillus subtilis,* were successfully detected, which suggested that spherical cells adhered more strongly to filter fibers than cylindrical cells [7].

Compared to conventional culture-dependent methods, metagenome sequencing methods (such as Illumina sequencing, Sanger sequencing, pyrosequencing, and NGS) and hybrid/chip technology methods can be used to identify microarrays of genomes present in atmospheric aerosol samples. These methods are highly sensitive, can be applied to any biological matter containing nucleic acids, and represent quick and dependable approaches for detecting the presence of both living and dead cells, as well as pathogenic and nonpathogenic microbes in the atmosphere [8]. The first metagenomics-based study performed by using the next-generation sequencing technique identified some predominant bacteria in the air, such as *Proteobacteria, Firmicutes, Actinobacteria*, unclassified *Enterobacteriaceae, Staphylococcus, Acinetobacter, Leuconostoc, Pseudomonas*, and *Lactobacillus*. Similarly, *Penicillium, Aspergillus, Rhizopus, Wallemia*, and *Hemicarpenteles* represented some of the predominant fungi detected [8,9]. Thus, the high-throughput sequencing method is a promising tool to explore bioaerosol diversity. Virus detection is difficult to achieve in the environment using a simple conventional method given the size and properties of viruses. As a result, metagenomics research has opened the door to effective virus identification. The sequences of polyomavirus, human papillomavirus, and other active viruses were identified in the metagenomics data from the cattle processing area as well as some indoor and some outdoor areas [10]. Another emerging tool that shapes microbial diagnosis is whole-cell mass spectrometry (WC-MS). This method has identified a wide range of bacteria, including Gram-positive and Gram-negative bacteria, as well as various classes of fungi with less time and effort [4].

Similarly, several real-time bioaerosol detection methods have also been identified and are being applied. For instance, the microoptofluidic platform the BioTrakTM Real-Time Viable Particle Counter and the BioLaz® Real-Time Microbial Monitor, which is a real-time electrostatic sampler useful for the collection, sizing, and enumeration of viable inhalable microbes present in the air, are two examples that are being used to monitor and detect bioaerosols [9,11]. These successful attempts have provided new information for understanding and performing further research with improved efficiencies and high accuracy, potentially offering innovations for bioaerosol measurements and controlling their impact on health and the environment.

## 3 Major issues of concern

**Fig. 2:**
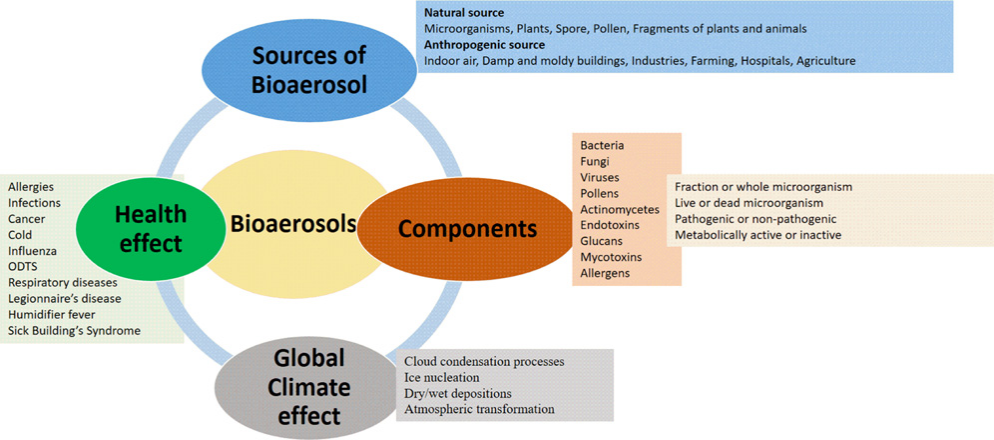
**Airborne bioaerosols: their sources, components, and their impact on health and the environment**.

The forthcoming competencies of the technologies discussed above fulfilling the limitations would shape the current potential for determining a reliable, efficient, easy, and fast method with maximum accuracy to accomplish identification and characterization of biological particles present in the atmosphere. Innovative and better systems for quantifying bacterial, fungal, and viral antigens, peptidases, proteases, and other influential enzymes must be developed in the future. The nature, source, biological and physical properties of bioaerosols are vast and require intensive study for better understanding (as shown in Fig. 2). A range of serious disease pathogens potentially exists in bioaerosols. However, the vital physical and biological causative agents for such illnesses remain vague. This limited knowledge is outwardly due to a lack of valid and accurate methods to assess those biological agents quantitatively. It is essential to take into consideration why some diseases are seasonal, such as seasonal flu, and some infections become epidemics or pandemics—for example, a debate on the transmission of COVID-19 through aerosols. Bioaerosol particles are generally 0.3–100 μm in diameter. In contrast, the respirable size fraction is approximately 1–10 μm. Particles with sizes ranging from 1.0 to 5.0 μm generally remain in the air for a longer time, whereas larger particles are deposited more rapidly on surfaces. The deposition of airborne particles depends on size, time and wind patterns [4]. Therefore, when an infected person coughs, sneezes, breathes vigorously, or speaks loudly, the virus is excreted and dissolves with the aerosol and becomes a bioaerosol with a particle size of approximately 1–5 mm, which can further spread in the space of approximately 1–2 m (the aerosol can travel hundreds of meters or more) [7]. It is important to note that previous research has confirmed that aerosols are involved in the spread of several respiratory diseases, such as influenza and aspergillosis [4]. COVID-19 may be transmitted through aerosols, but it requires further verification by experiments.

Several other diseases that may be spread through aerosols have potentially not been reported. Hence, extensive studies and experimental research are required to identify the source, type, and pathogenicity of bioaerosols in human health. Sharma Ghimire et al. (2019) briefly discussed essential future acts that need to be applied in the field of bioaerosol research [9]. Atmospheric transference and ecological interaction with bioaerosols are also promising avenues for future investigation, as they are correlated and could provide valuable information about some undefined parameters and advance research on the role of microorganisms in health and the environment. Indeed, an advanced detection system requires equipment designed with specific features, such as a high-capacity power backup, high-quality radiation, high specific wavelengths, high-quality light detection systems, and high-quality laser sources for better performance. Similarly, the interaction of biological agents with chemical and physical agents in the development of diseases and symptoms is another important concern that needs to be addressed.

On the other hand, the actual progress of this era involves instruments that should be smaller, portable, cheaper, and easier to use, yet faster, more accurate, and more reliable. For example, focusing on the impact of bioaerosols on health and the environment is essential in sensitive areas, such as the Polar region and the Hindu Kush-Himalayas (HKH), which is also known as the Third Pole, and economically developing countries. The remoteness and synoptic atmospheric patterns make polar regions geographically isolated areas that are mainly characterized by changes in albedo, sea-ice extent, ice sheet melt, and glacial retreats, which greatly affect the polar radiation budgets. In addition, the melting of terrestrial ice creates new sites for microbial colonization [2]. The extreme weather conditions and logistics in those areas make regular sampling and sample maintenance difficult. Furthermore, due to the region's severe conditions, it is extremely difficult to obtain reliable samples and data. Similarly, Asian dust episodes are a predominant phenomenon of soil-derived dust being transported over long distances across large sections of the HKH and Tibetan Plateau (TP) regions, East Asia, and even reaching Arctic regions [9,12]. A third pole region covering the Himalayas and the TP contains high elevation areas with harsh environmental conditions that pose difficulties in sampling due to lack of transport and logistics, making these regions challenging for such research. As a result, these regions are of particular importance to aerobiology in the present context. However, the size, cost, and scarcity of advanced techniques and expertise hinder research in these areas.

## 4 An innovative approach that involves a combination of techniques is a crucial need

**Fig. 3:**
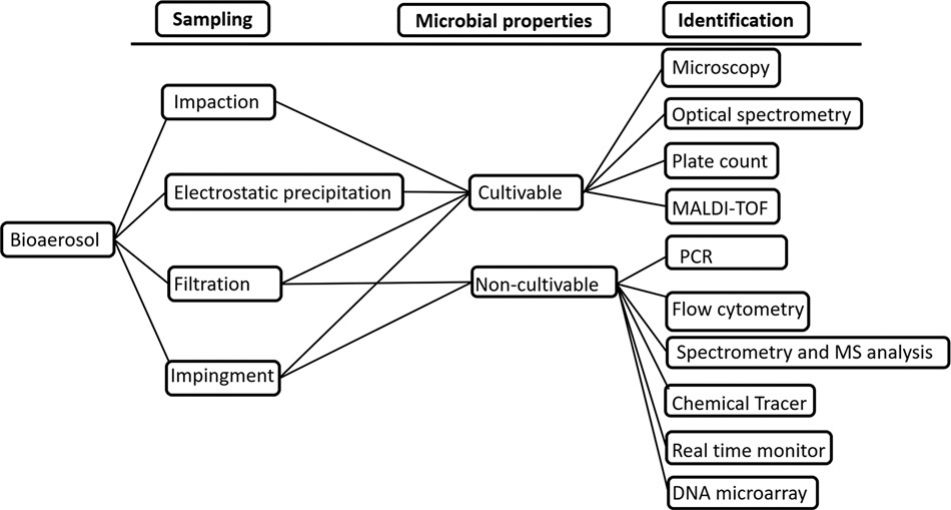
**Sampling and identification methods that can be combined and applied for bioaerosol study**.

One of the most critical gaps in bioaerosol science is the lack of coupled details of biological analyses, culture characteristics, genomic, proteomic, and metabolomics approaches, and real-time data (Fig. 3). In the past decade, several studies have been published using techniques focusing on molecular and isotopic indicators for tracing bioaerosol particles released from various sources in the environment, such as next-generation sequencing and laser/fluorescence-induced spectroscopy [4]. However, the absence of an accurate, rapid, easy, and inexpensive method for quantifying bioaerosols remains a barrier to assessing biological aerosol concentrations and their health effects. The difficulty persists from sample collection until the analytical procedure. Principally, the sample collection procedures for microorganisms and other particles are similar and are primarily based on filtration, impaction, or liquid impingement. Numerous other factors plague the measurement and analysis of bioaerosol-related parameters, such as sampling inlet, particle exclusion, biological recovery, growth and survival of the individual organism, assay efficiencies, humidity, temperature and pH of the culture medium. Furthermore, the selection of proper sampling and identification media and even accuracy in microscopic observation are other factors that interfere with obtaining appropriate results. Moreover, the connection among the quantitative characterization of bioaerosols present on the surface, planetary boundary layer, and troposphere represents another problem to attaining the overall (vertical and horizontal) circulation of biological particles [1]. Similarly, a portable device has also been devised that can be carried in any particular area (such as residential areas, sterile areas, dumping sites, and hospitals) to identify and characterize microbes present in the air in real-time [4]. For instance, as explained by Saikai et al. (2015), for culturable microorganisms, a device with a culturable chamber has been developed that has the ability to incorporate growth media, maintain temperature and measure the abundance and biochemical properties of microorganisms [1]. Other methods may include a high magnification lens to detect the type of living microorganisms with the camera sensor and for mapping the structure, size, characteristics of microorganisms or biofilms and a device with a real-time sequence analyzer in a specific volume of air to identify the microorganisms [4]. Similarly, a data storage system for microbial diversity as well as the setup of the above discussed real-time microbial identification device that can assist in maintaining remote sensing study of microbes in the air represents another innovative method to explore bioaerosols [1]. To report these concerns, the results from real-time detection, NGS sequencing, mass analysis, and biological and chemical analysis can be compared with climatic studies. These data can be mathematically modeled to predict conditions and interactions in Earth's history and future climate. It is crucial to strengthen teamwork among interdisciplinary areas of biology, chemistry, earth science, and life science through shared understanding and knowledge among researchers and experts worldwide. *Science* and *Astronomy* published a study by Elizabeth Howell on April 15, 2019, revealing that bacteria and fungi are present all over space. Similarly, Capone and Subramaniam (2015) [13] performed an outstanding study on the use of remote sensing as a resource for tracking marine microbial ecosystem dynamics. These discoveries are advancing our knowledge. If combined with other stable and standard techniques, bioaerosol investigation will substantially improve the pace of our research. The above explanation concurrently suggests that bioaerosol studies will be much advanced and accurate if a simple yet functionally sophisticated device can be used anywhere on the Earth's surface. These creative ideas may appear unrealistic at present, but nothing is impossible in the field of science and technology.

## 5 Conclusion

Future challenges, such as climate change, health deterioration, global economic downturns, and an increase in airborne pathogens, are likely due to the negative environmental and social effects of bioaerosols. Hence, a vast amount of investigation is crucial to identify what is present in the air and poses a negative impact on the environment and climate. This short article provides a broad concept of analytical opportunities for researchers, such as conventional culture methods followed by gene-based studies, metagenomics studies combined with molecular studies, and mass spectrometric analysis together with biochemical analysis, which could deliver significant amounts of information about the microbial processes occurring in the environment. No single aerosol sampling and measurement method is expected to be appropriate for all prospects (size, species, and specific research hypotheses). These limitations can be overcome by using cross-disciplinary strategies with the combined use of several analytical methods that can exclude possible errors. This perspective has attempted to provide new concepts or designs to untangle some innovative possibilities for future research.

## Acknowledgments

This work was supported by China Postdoctoral Science Funding (Grant No. 2019M663859). The second Tibetan Plateau Scientific Expedition and Research Program (STEP) (Grant No. 2019QZKK0605).

## Declaration of Competing Interest

The authors declare that they have no conflicts of interest in this work.
